# Lighting Up the Tumor—Fluorescein-Guided Resection of Gangliogliomas

**DOI:** 10.3390/jcm9082405

**Published:** 2020-07-28

**Authors:** Julius Höhne, Francesco Acerbi, Jacopo Falco, Mehmet Osman Akçakaya, Nils Ole Schmidt, Talat Kiris, Camilla de Laurentis, Paolo Ferroli, Morgan Broggi, Karl-Michael Schebesch

**Affiliations:** 1 Department of Neurosurgery, University Medical Center Regensburg, 93053 Regensburg, Germany; nils-ole.schmidt@ukr.de (N.O.S.); karl-michael.schebesch@ukr.de (K.-M.S.); 2 Department of Neurosurgery, Fondazione IRCCS Istituto Neurologico Carlo Besta, 20133 Milano, Italy; Francesco.Acerbi@istituto-besta.it (F.A.); jacopo.falco@istituto-besta.it (J.F.); camilla.delaurentis@istituto-besta.it (C.d.L.); paolo.ferroli@istituto-besta.it (P.F.); morgan.broggi@istituto-besta.it (M.B.); 3 Department of Neurosurgery Liv Hospital Ulus Affiliated with Istinye University Medical Faculty, Istanbul 34340, Turkey; moakcakaya@gmail.com (M.O.A.); talatkrs@gmail.com (T.K.)

**Keywords:** ganglioglioma, fluorescein sodium, YELLOW 560 nm filter, fluorescence-guided surgery, PENTERO 900, KINEVO, surgical microscope, neurosurgery

## Abstract

(1) Background: Gangliogliomas comprise a small number of brain tumors. They usually present as World Health Organization (WHO) grade I, and they delineate on gadolinium-enhanced MRI; the surgical goal is wide radical resection, and the course thereafter is usually benign. Fluorescein sodium (FL) tends to accumulate in areas with altered blood–brain barrier (BBB). Thus far, the results provided by prospective and retrospective studies show that the utilization of this fluorophore may be associated with better visualization and improvement of resection for several tumors of the central nervous system. In this study, we retrospectively studied the effect of fluorescein sodium on visualization and resection of gangliogliomas. (2) Methods: Surgical databases in three neurosurgical departments (Regensburg University Hospital; Besta Institute, Milano, Italy; and Liv Hospital, Istanbul, Turkey), with approval by the local ethics committee, were retrospectively reviewed to find gangliogliomas surgically removed by a fluorescein-guided technique by the aid of a dedicated filter on the surgical microscope from April 2014 to February 2020. Eighteen patients (13 women, 5 men; mean age 22.9 years, range 3 to 78 years) underwent surgical treatment for gangliogliomas during 19 operations. Fluorescein was intravenously injected (5 mg/kg) after general anesthesia induction. Tumors were removed using a microsurgical technique with the YELLOW 560 Filter (YE560) (KINEVO/PENTERO 900, Carl Zeiss Meditec, Oberkochen, Germany). (3) Results: No side effects related to fluorescein occurred. In all tumors, contrast enhancement on preoperative MRI correlated with bright yellow fluorescence during the surgical procedure (17 gangliogliomas WHO grade I, 1 ganglioglioma WHO grade II). Fluorescein was considered helpful by the operating surgeon in distinguishing tumors from viable tissue in all cases (100%). Biopsy was intended in two operations, and subtotal resection was intended in one operation. In all other operations considered preoperatively eligible, gross total resection (GTR) was achieved in 12 out of 16 (75%) instances. (4) Conclusions: The use of FL and YE560 is a readily available method for safe fluorescence-guided tumor resection, possibly visualizing tumor margins intraoperatively similar to contrast enhancement in T1-weighted MRI. Our data suggested a positive effect of fluorescein-guided surgery on intraoperative visualization and extent of resection during resection of gangliogliomas.

## 1. Introduction

Gangliogliomas comprise a small number of brain tumors [1]. They are composed of ganglion cells (neurons) and glial (astrocytic) cells, and hence, according to predominance, they are classified into ganglioneuromas and gangliogliomas. Courville coined the term “ganglioglioma” in 1930 [2]. They are slow-growing, well-differentiated, and usually present as World Health Organization (WHO) grade I [3]. In 1979, the WHO classified gangliogliomas, a subtype of low-grade gliomas (LGG), as grade I or II, since malignant transformation may occur. Rare cases with anaplastic features are acknowledged as grade III or IV [4]. Sporadically, as described by case reports, metastatic behavior may occur [5]. The incidence of gangliogliomas is reported to be between 0.4% and 7.6% of brain tumors [1,6,7]. Because of their low incidence, algorithms for diagnosis and treatment stem from single-center reports and case reports. Patients with gangliogliomas are usually young, and seizures are typically the presenting symptom, which are difficult to control medically [8]. Furthermore, the neoplasm is most commonly related to temporal lobe epilepsy and can occur in patients with neurofibromatosis types I and II and Turcot syndrome [9]. The majority of tumors are located above the tentorium and often based cortically.

Gangliogliomas show high heterogeneity in imaging studies. Calcification has been described on plain skull X-ray and computed tomography (CT), on magnetic resonance imaging (MRI), high T1-weighted imaging (T1WI), short-tau inversion recovery (STIR), and fluid-attenuated inversion recovery (FLAIR) signal. Low T2-weighted imaging (T2WI) signal is common, with no diffusion restriction on diffusion-weighted imaging (DWI) and apparent diffusion coefficient (ACD). Calcification may become visible on T2*/gradient echo sequences (GRE)/susceptibility weighted imaging (SWI) sequences. Contrast enhancement is a common feature [5,10].

The surgical goal is wide radical resection, depending on the eloquence of the tissue involved, and the course thereafter usually benign [8,11]. In one series, the reported survival was 89% and 84% at 5 and 10 years, respectively, for WHO grade I and 83% at 10 years for WHO grade II–III [11]. Patients are usually followed up by regular imaging, as re-resection is advised at recurrence. Due to its long-term benign course, radiation therapy is generally considered in selected cases at recurrence. Palliative care with chemotherapy, radiation therapy, or both is an option for unresectable tumors [12].

Apart from standard microsurgical resection utilizing the operating microscope, 5-aminolevulinic acid (5-ALA) fluorescence has been evaluated as an intraoperative tool for improved visualization with limited to no benefit [13,14,15]. Near-infrared optical imaging in one study could visualize tumors with preoperative gadolinium enhancement on MRI [16]. Since most gangliogliomas appear on imaging studies with some form of contrast enhancement [10], this finding is indicative of a pathological alteration of the blood–brain barrier (BBB). Recent retrospective and prospective studies suggest that the fluorophore fluorescein sodium (FL) improves visualization and resection of a plethora of central nervous system tumors [17,18,19,20,21,22,23]. Passive staining of the extracellular space in areas with a BBB, corresponding to the gadolinium uptake on MRI [24], is the presumed mechanism of action. Because even single perivascular glioma cells disrupt the BBB [25,26], it stands to reason that this effect could improve glioma resection. The safety of this fluorophore has been demonstrated with more than 40 years of experience in ophthalmology, a field where it is used as a vascular dye [27].

Here, we present an alternative technique to localize and examine gangliogliomas intraoperatively, ultimately guiding tumor resection, using FL and an FL-specific microscope filter. This multicenter study, to our knowledge, is the first report on the effect of FL on visualization and resection of ganglioglioma.

## 2. Experimental Section

### 2.1. Patients and Pre- and Postoperative Clinical and Radiological Evaluation

In this study, we systematically and retrospectively reviewed surgical and neuropathological databases at three neurosurgical departments (Department of Neurosurgery, University Medical Center Regensburg, Regensburg, Germany; Fondazione I.R.C.C.S. Istituto Neurologico Carlo Besta, Milan, Italy; Department of Neurosurgery Liv Hospital Ulus affiliated with Istinye University Medical Faculty, Istanbul, Turkey) to identify the cohort of patients with ganglioglioma who had undergone fluorescence-guided tumor resection with fluorescein sodium (FL) at any of the centers between May 2012 and February 2020. Inclusion criteria were informed consent about the off-label use of FL, histopathologically confirmed ganglioglioma, and no known allergy to FL. The retrospective study was approved by the respective institutional review boards.

We identified 18 patients (13 women, 5 men; mean age 22.9 years, range 3 to 78 years) with gangliogliomas, including 15 gangliogliomas classified as WHO grade I, 1 desmoplastic infantile ganglioglioma classified as WHO grade I, one ganglioglioma/subependymal giant cell astrocytoma classified as WHO grade I, and one anaplastic ganglioglioma classified as WHO grade II. All patients underwent preoperative and prompt postoperative (within 72 h after surgery) contrast-enhanced MRI. A review of preoperative imaging determined localization and contrast-enhancing behavior of the tumor. The extent of resection was estimated by a neuroradiologist comparing pre- and postoperative imaging.

Data on the clinical and neurologic condition at admission and discharge were available for all patients (Table 1).

### 2.2. Surgical Protocol

Following reports published earlier [18,28], a weight-adjusted dose (5 mg/kg body weight) of FL (Alcon Pharma, Freiburg im Breisgau, Germany; Monico S.p.A., Venice, Italy; Alcon Laboratuvarları, Istanbul, Turkey) was injected intraoperatively after a central venous line was established at the induction of anesthesia, approximately 30–45 min before approaching the lesion. Subsequently, tumorous tissue was then removed with the aid of FL-induced fluorescence visualized with an additional filter on the operating microscope (KINEVO 900 or PENTERO 900, ZEISS Meditec, Germany). This filter (YELLOW 560) is tailored to the excitation and emission wavelength of FL and allows for improved visualization and dose reduction [29]. When applicable, the Cavitron ultrasonic surgical aspirator (CUSA) was used. Typically, in FL-guided surgery, the lesion exhibits bright yellow staining when approached. Resection was stopped when the yellow-green staining of the enhancing tissue became faint and pinkish nonenhancing tissue appeared at the circumference of the tumor. Naturally FL-enhancing areas, such as the ventricular wall, were carefully identified. Unless continuation of surgery was deemed unsafe, for instance, because of venturing into eloquent areas, surgical intervention was finished after removal of all fluorescing tissue as confirmed by filter view. Intraoperative monitoring, intraoperative ultrasound, and neuronavigation were used in selected cases. For one patient, an awake craniotomy was conducted due to speech eloquent localization of the tumor (Pat. 4, Table 1). 

### 2.3. Preoperative Use of Glucocorticoids

Due to differences in institutional standards, four patients received glucocorticoids immediately preoperatively (Pat. 1–4, Table 1). Another patient (Pat. 16) was preoperatively on a regular dose of glucocorticoids for symptom relief.

### 2.4. Intraoperative Fluorescence Characteristics and Side Effects

Surgical reports were screened for subjective evaluation of the grade of fluorescent staining of the targeted lesion. The screening was conducted for any reference to the degree of fluorescent staining: “bright” versus “medium and heterogeneous” versus “effectively no fluorescence”. Furthermore, medical reports were evaluated for any possible adverse effect or allergic reaction to FL.

### 2.5. Extent of Resection

Gross total resection (GTR) was defined as no residual contrast on postoperative MRI, near-total resection (NTR) was defined as less than 1 cm^3^ contrast enhancement, and subtotal resection (STR) was defined as more than 1 cm^3^ of contrast enhancement. Furthermore, cases were stratified by whether an identifiable tumor was left behind, due to anatomical or functional considerations intraoperatively, as well as by whether there was an intended biopsy.

### 2.6. Ethical Approval

All procedures conducted in studies involving human participants were in accordance with the ethical standards of the institutional and/or national research committee and with the 1964 Helsinki Declaration and its later amendments or comparable ethical standards.

## 3. Results

### 3.1. Fluorescence

In all patients (*n* = 18; 100%), intense homogenous or heterogeneous yellow-green fluorescent staining of tumor tissue was noted. In all cases but one (Pat. 8), intraoperative fluorescence was deemed helpful for improved resection. In the one case, fluorescent guidance was not necessary due to good localization of the lesion and intended STR. No technical difficulties regarding the use of the microscope filter were encountered during the surgical interventions.

### 3.2. Extent of Resection

In all lesions, contrast enhancement was noted on preoperative MRI. In a total of 19 operations, GTR, according to no residual contrast enhancement, was achieved in 12 operations (67%). Residual tumor tissue was seen on postoperative MRI in seven cases (33%). In those seven patients, a biopsy was intended in two operations (Pat. 7), and subtotal resection was intended in one (Pat. 8, sellar and suprasellar tumor debulking). In three patients (Pat. 13, 17, and 18), GTR was deemed possible according to the preoperative MRI, but intraoperative constraints (anatomical or functional considerations) limited the extent of resection (EOR). In one patient (Pat. 1), residual contrast enhancement was noted on the postoperative MRI. 

### 3.3. Karnofsky Performance Scale (KPS)

At baseline, 15/18 (83%) of patients had KPS scores of 90–100, indicating good clinical and neurological conditions. Surgical morbidity led to a postoperative decline of KPS score in five patients at discharge. No long-term follow-up data were available; such data were not relevant to the scope of this study, as we solely focused on the quality of resection validated by early postoperative MRI.

### 3.4. Adverse Events

We did not encounter any morbidity or mortality attributable to the use of FL. Furthermore, no major side effects related to fluorescein throughout the observation period apart from yellow-colored urine and, in some patients, slight yellow discoloration of the skin were documented.

### 3.5. Representative Case

**Figure 1:**
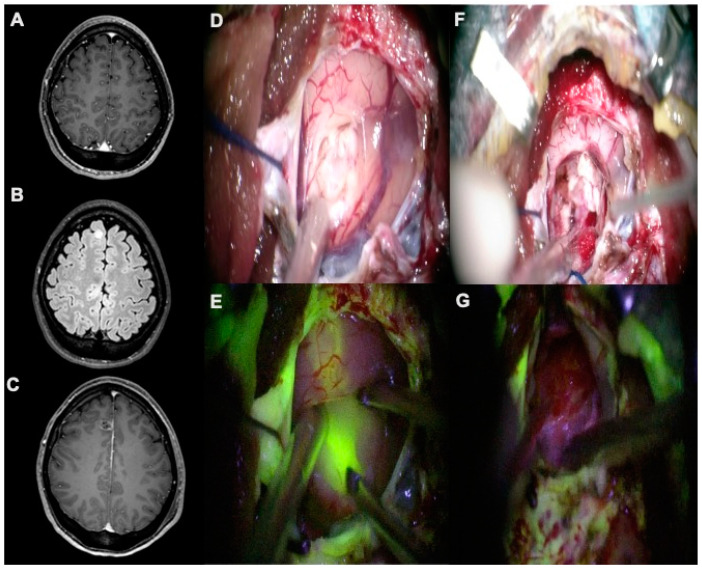
(**A**,**B**) Preoperative, postcontrast axial T1-weighted and axial FLAIR-weighted scans showing a right frontomesial ganglioglioma, completely removed as detectable by postoperative, postcontrast axial T1-weighted scan (**C**). (**D**,**E**) During surgical resection, YELLOW 560 filter highlighted pathologic tissue allowing the identification of a clear plane of dissection by using the fluorescent visualization. (**F**,**G**) At the end of resection, the cavity did not show any sign of a residual tumor, as confirmed by fluorescent visualization with YELLOW 560 filter.

Figure 1 represents intraoperative photographs of a 19-year old female that presented with seizures. Preoperative postcontrast T1-weighted imaging revealed a right frontomesial, circular enhancing lesion, which was resected by FL guidance. A supplementary intraoperative video can be found online.

## 4. Discussion

Despite the technical advancements, many surgeries of different lesions of the central nervous system (CNS) still solely rely on visual cues and tactile differentiation when resecting tumorous tissue. To name a few, the operating microscope, gadolinium-enhanced MRI, ultrasound, and neuronavigation substantially extended the armamentarium available to neurosurgeons over the past decades to safely aid maximal EOR. Fluorescence-guided surgery, fitted with the operating microscope, has been part of this evolution. Apart from its off-label use in detecting cerebrospinal fluid leaks, FL has regained interest amongst the neurosurgical community in the past decade. It has been shown to safely guide tumor resection in numerous tumors of the CNS, including but not limited to high-grade gliomas, metastases, and hemangioblastomas [17,18,19,20,21]. FL fluorescence can be observed by the naked human eye at high doses [30], but the addition of a specific filter has allowed for a significant dose reduction, lowering the odds of dose-related complications. Furthermore, good image quality that depicts anatomy in high detail allows for continued microsurgical work in filter mode, an advantage not seen with other fluorescent agents like indocyanine green (ICG) or 5-ALA. ICG has not been evaluated for ganglioglioma surgery. Use of the fluorophore 5-ALA has been reported, but the level of evidence is poor. In a study by Valdes et al. in which 5-ALA was evaluated in 12 cases of LGG, including two gangliogliomas, faint visual and quantitative probe-based fluorescence could only be detected in one of the two [14]. In another series with 27 LGG, including one desmoplastic infantile ganglioglioma, moderate diffuse fluorescence was reported by Goryaynov et al., using a slightly higher 5-ALA dose (25 mg/kg) [15]. Another retrospective study by Preuss et al. evaluating 5-ALA in 18 pediatric patients with suspected brain tumors found minimal positive staining of the internal parts of the tumor but no enhancement of the tumor in one of three gangliogliomas [13]. Minkin et al. evaluated high-dose FL in 11 patients with benign neuroepithelial primary brain tumors, including two gangliogliomas, using unfiltered microscope lighting and found it helpful for tumor delineation [31]. In our study, FL was utilized to help with visualization and maximize resection. The dosage of FL followed that used in earlier studies [17,21]. The recent evaluations of FL did not include any reports on relevant side effects, apart from yellowish discoloration of the skin, scleras, and urine, which is usually fully reversible within 48 h. FL has been extensively evaluated in ophthalmology, and very few adverse events have been registered during intracranial surgery [32,33].

To our knowledge, this is the first report evaluating low-dose FL in ganglioglioma surgery. The use of FL could improve tumor visualization and resection in this entity. Moreover, our observation was associated with gadolinium enhancement on preoperative MRI, which was present in all tumors in our series. Surgeon-reported visibility of tumorous tissue was clearly enhanced by FL and filter view and almost always considered helpful for complete tumor removal.

The goal of GTR was achieved in 12 out of 16 (75%) operations when GTR was preoperatively considered possible and intended. In 3 out of 16 (19%) of the remaining operations, residual tumor tissue was left behind intentionally to preserve function or limit surgical morbidity, and in 1 out of 16 (6%) operations, residual contrast enhancement was noted on the postoperative MRI. In a study of Varshneya et al., of 198 adult patients with low and high-grade gangliogliomas, the authors found overall gross total resection rate of 59% [34]. Of 348 children with low-grade ganglioglioma or gangliocytoma in a Surveillance, Epidemiology, and End Results (SEER) Database study by Dudley et al., surgery was performed on 91.6% of cases, with GTR achieved in 68.3% [7]. The 67% rate of GTR across centers in our study is comparable. As with every other fluorophore, the added benefit relies on adequate exposure to visualize fluorescence. The reasons for incomplete resection, even in tumors that are preoperatively deemed suitable for GTR, are manifold. Despite positive fluorescence, the proximity to eloquent tissue or adherence to vessels can be misleading on preoperative imaging. Other reasons are inadequacy of the surgical window or sagging of the overlying brain into the cavity. Although all tumors in this series were contrast enhancing, areas that were not contrast enhancing could have been fluorescence negative, thus evading proper intraoperative identification.

No adversaries to the surgical workflow or ergonomics were encountered. In line with earlier experiences using FL for other intracranial pathologies, no side effects attributable to the use of FL occurred intraoperatively or during the hospital stay.

The exact mechanism by which FL accumulates in gangliogliomas is not yet known. This holds for other pathologies of the CNS, evaluated with FL. The striking mutual feature is gadolinium enhancement, indicating an impairment of the BBB and likely accounting for the FL staining, as both are distributed via the same route. Glioma cells can precipitate the breakdown of the blood–brain barrier (BBB), which could enable FL to permeate into the extracellular space, possibly facilitating fluorescence [24,25,35]. Assuming this to be true, FL could be a marker of the tumor path into the healthy brain. Neira et al. found intraoperative FL staining to be equivalent to gadolinium uptake and even to extend beyond the gadolinium-enhancing region, possibly due to its lower molecular weight [36]. Furthermore, the authors could show a high positive predictive value of fluorescent staining for identifying glioma tissue. Concerning cost-effectiveness, a meta-analysis by Eljamel et al. showed that the incremental cost per quality-adjusted life-year with FL-guided surgery in high-grade glioma surgery was significantly lower compared with intraoperative ultrasound, intraoperative MRI, and 5-ALA [37].

Although intraoperative evaluation of the fluorescence was subjective in this study, the report of helpful fluorescence in almost all cases, particularly at the tumor margins, taken together with a high rate of GTR, suggests a reproducible effect. FL is still undergoing feasibility tests, particularly in combination with the YELLOW 560 nm microscope filter. Final approval of the drug by the respective competent authorities is pending. In Italy, hospitals are reimbursed for the usage of the drug.

Optimal dosage and timing of FL in ganglioglioma surgery, as well as its role in redo surgery, is not known. Although not all gangliogliomas are contrast enhancing on preoperative MRI [5,10], all tumors in this series were. Therefore, the effect of FL on the resection of nonenhancing gangliogliomas needs to be elucidated. Furthermore, the effect of FL on recurrence-free and overall survival is not known. This needs to be evaluated in a randomized, controlled clinical trial with adequate power to precisely assess the outcomes within a predefined observation period.

## 5. Conclusions

Our data suggested a positive effect of fluorescein-guided surgery during resection of gangliogliomas with contrast enhancement on preoperative MRI. We conclude that FL and the YELLOW 560 nm filter are safe and feasible tools. The use of FL and YELLOW 560 nm filter is a readily available method for fluorescence-guided tumor resection, possibly visualizing tumor margins intraoperatively similar to contrast enhancement in T1-weighted MRI.

## Figures and Tables

**Table 1:** Characteristics of the patients and main results.

Pat.	Age	Sex	Location	Presenting Symptom	EOR	FLI	Histology	KPS Pre	KPS Post
1	52	f	Left frontal	Headache	STR	++	Ganglioglioma (WHO grade I)	100	100
2	21	f	Left frontal	Seizure	GTR	++	Ganglioglioma (WHO grade I)	100	100
3	32	f	Left temporal	Headache	GTR	++	Ganglioglioma (WHO grade I)	100	100
4	37	m	Left temporal	Expressive language disorder	GTR	++	Ganglioglioma (WHO grade I)	100	100
5	19	f	Right frontal	Seizure	GTR	+	Ganglioglioma (WHO grade I)	100	100
6	55	m	Left parieto-occipital	Loss of consciousness	GTR	++	Ganglioglioma (WHO grade I)	100	100
7	3	f	Medulla oblongata	Trigeminal pain	NTR Biopsy	+	Ganglioglioma (WHO grade I)	100	60
8	8	m	Sellar and suprasellar	Visual disturbance, hormonal deficit	STR (i)	+	Ganglioglioma (WHO grade I)	70	70
9	16	m	Left frontal	Seizures	GTR	+	Ganglioglioma (WHO grade I)	100	100
10	78	f	Left temporal	Expressive language disorder	GTR	++	Anaplastic ganglioglioma (WHO grade II)	90	30
11	20	f	Right frontal	Seizure	GTR	++	Ganglioglioma (WHO grade I)	90	90
12	17	f	Left temporal	Seizure, cognitive deficit	GTR	++	Ganglioglioma and subependymal giant cell astrocytoma (WHO grade I)	80	80
13	3	f	Right temporal	Abnormal head posture	NTR (i)	+	Ganglioglioma (WHO grade I)	90	90
14	16	f	Left temporoparietal	Seizure	GTR	+	Ganglioglioma (WHO grade I)	90	80
15	15	f	Left parietal	Follow-up in NF 1	GTR	++	Ganglioglioma (WHO grade I)	100	100
7	4	f	Multifocal right temporal Biopsy	Follow-up (Patient 7)	Biopsy	++	Ganglioglioma (WHO grade I)	90	90
16	14	m	Right parietal	Seizure	GTR	++	Ganglioglioma (WHO grade I)	90	90
17	3	f	Vermis, cerebellar	Ataxia	STR (i)	++	Desmoplastic infantile ganglioglioma (WHO grade I)	80	60
18	23	f	Cerebellar, vermis, midbrain, pineal	Headache	NTR (i)	+	Ganglioglioma (WHO grade I)	100	70

Demographic data, all patients received 5 mg/kg fluorescein at induction of anesthesia; no side effects encountered; Pat. = patient, sex: m = male, f = female; EOR = extent of resection (GTR = gross total resection, STR = subtotal resection, NTR = near total resection, i = intended); FLI = fluorescence intensity (++ bright, + medium); KPS = Karnofsky Performance Scale.

## Supplementary Materials

The following are available online at https://www.mdpi.com/2077-0383/9/8/2405/s1, Video S1: Fluorescein-guided resection of a right frontomesial ganglioglioma.
